# Structural Biology: A Century-long Journey into an Unseen World

**DOI:** 10.1179/0308018815Z.000000000120

**Published:** 2015-12-07

**Authors:** Stephen Curry

**Affiliations:** ^a^ Department of Life Sciences, Imperial College London, UK

**Keywords:** X-ray crystallography, nuclear magnetic resonance spectroscopy, solution scattering, electron microscopy, biological macromolecules

## Abstract

When the first atomic structures of salt crystals were determined by the Braggs in 1912–1913, the analytical power of X-ray crystallography was immediately evident. Within a few decades the technique was being applied to the more complex molecules of chemistry and biology and is rightly regarded as the foundation stone of structural biology, a field that emerged in the 1950s when X-ray diffraction analysis revealed the atomic architecture of DNA and protein molecules. Since then the toolbox of structural biology has been augmented by other physical techniques, including nuclear magnetic resonance spectroscopy, electron microscopy, and solution scattering of X-rays and neutrons. Together these have transformed our understanding of the molecular basis of life. Here I review the major and most recent developments in structural biology that have brought us to the threshold of a landscape of astonishing molecular complexity.

## Introduction

When Orville Wright took off in the *Flyer* on a grey morning in December 1903 and flew for all of twelve seconds across the sands near Kitty Hawk in North Carolina, little could he have suspected that by 1969 powered flight would land Neil Armstrong and Buzz Aldrin on the Moon. Humankind’s first foray onto another world remains for many people one of the greatest technological achievements of the twentieth century. But within the sixy-six years it took to get from Kitty Hawk to Tranquillity Base another equally remarkable technological — and scientific — journey took place, one that has brought us to a very different destination.

In 1912, from experiments initiated by Max von Laue in Germany and successfully analysed by William and Lawrence Bragg in England, X-rays were first used to peer into the atomic structure of crystalline matter. By the end of the 1950s X-ray crystallography had leapt from physics to chemistry to biology and the atomic architecture of DNA and several proteins had been revealed, giving us the first glimpses of a molecular landscape that was no less surprising and no less strange than the surface of the Moon. It had taken just five decades for structural biology to emerge as a fledgling discipline. In the five that have since elapsed the field has grown vigorously, thanks not only to developments in X-ray crystallography but also to the emergence of complementary techniques that have used other physical phenomena to lift the veil on an unseen world — the atomic and molecular matrix of life.

Until the twentieth century, that world was invisible because it was out of reach of the light microscope. The limitations of this instrument had been defined in 1873 by Ernst Abbe, who realized that the finite dimensions of its lenses and apertures would give rise to diffraction — the optical phenomenon that a beam of light spreads out as it passes through a narrow opening. This means that a point source would invariably be imaged as a diffuse disc of light; as a result the image formed of any object made up of fine points is blurred and loses definition.

Abbe showed that with visible light, which has a wavelength of around 0.5 µm (or 0.0005 mm), only objects larger than half the wavelength can be resolved from one another. This puts bacterial cells (~1 µm) just within reach. Animal and plant cells, which are typically 10–100 µm across can be resolved, along with sub-cellular organelles such as nuclei (~5 µm) and mitochondria (0.5–10 µm), though their finer details cannot be discerned. Microscopy offers no hope of peering into the structures that cells are mostly made of — the protein, DNA, RNA, carbohydrate, and lipid macromolecules (typically 0.002–0.050 µm) — never mind their component atoms, which are around 0.0001 µm (= 0.1 nm or 1 Ångstrom (Å)) in diameter.

X-rays, which lie on the same spectrum of electromagnetic radiation as visible light, have much smaller wavelengths (around 0.1 nm). In principle, they could be used image molecules in atomic detail but the materials do not yet exist to build an effective X-ray microscope (Schneider 2003). However, in 1912 Laue showed that X-rays could provide a radical new way of imaging the sub-structure of the world. In an experiment originally designed to demonstrate their wave nature, Laue’s co-workers, Friedrich and Knipping, fired a fine beam of X-rays at a crystal of copper sulphate and recorded a pattern of regular spots on a photographic plate: the ordered, close-packed array of atoms within the crystal had caused the incident beam to diffract into many directions (as expected if X-rays were waves). The resulting diffraction pattern was nothing like a microscopic image but hinted at the atomic structure within. At the end of that year, twenty-two year-old Lawrence Bragg, who had been shown Laue’s data by his father William, realized in a moment of brilliant insight that treating the crystalline diffraction as the reflection of X-rays from the planes of atoms within the crystal lattice allowed the atomic positions to be deduced. This was a key breakthrough and the Braggs quickly established X-ray crystallography as a powerful tool for structural analysis by physicists, chemists, and biologists.

In this review I want to focus on the techniques that have particularly energized the field of structural biology, which first emerged in the 1930s thanks to the work of many scientists encouraged and inspired by the Braggs. It has diversified well beyond X-ray analysis as new techniques based on magnetism, the electron and the neutron have been developed that now complement and compete with crystallography in the quest to reveal the atomic structures of biological molecules in living cells. As with X-ray crystallography, each of these techniques embodies an indirect and highly processed approach to imaging and raises questions about what is actually being seen. Nevertheless, the methods that have been developed are undeniably powerful. They have brought a structural perspective that has greatly informed biochemistry and biology and helped to spawn new fields such biotechnology, bioinformatics, and synthetic biology as our ability to ‘see’ and study life at the molecular level has engendered new ideas, new tools, and even new industries.

Despite the progress of the last century, we shall find that we are still a long way from being all-seeing. Structural biology remains at heart a pragmatic science; it frequently has to face its own limitations, one of the most common being that molecular structures usually have to be solved in isolation or, at best, in complexes with a small number of partners. We are not yet able to observe the whole machinery of life in operation. But as we crossed the threshold of the twenty-first century, light microscopy is making a comeback with new super-resolution techniques that defy Abbe and are beginning to offer the hope that the molecules purified for analysis in structural biology laboratories might soon be imaged in their natural habitats.

## Biological X-ray crystallography

The development of biological crystallography is discussed in more detail elsewhere in this issue (see Brooks-Bartlett and Garman) so I will touch on it only briefly here to set the context for understanding the significance of other structure determination methods that have come in its wake.

The Braggs’ initial approach to the interpretation of diffraction patterns involved a degree of trial and error in using the information encoded in the diffraction spot positions to figure out the repeating arrangement of atoms within crystal lattices but was successfully applied to a number of salts, minerals and gemstones in the years following 1912. However, as early as 1915 William Bragg noted that application of the method to more complex compounds and structures would also require quantitative measurements of spot intensities and mathematical tools such as those developed by Fourier for analysing periodic systems (Bragg 1915). These provide the formalism required to relate positions and intensities of diffraction spots to the array of molecular structures within a crystal. It took William Bragg’s invention of the diffractometer, which, unlike photographic film, could give quantitative readouts of spot intensities before Fourier methods were used successfully to calculate the ‘appearance’ of atoms in the crystal, first in one dimension and then in two (Bragg 1929). Since X-rays are mostly diffracted by the electron clouds surrounding atomic nuclei, the method effectively yields maps of electron density; with good quality crystals these maps are sufficient to define atomic positions and so delineate molecular conformations.

**Figure 1:**
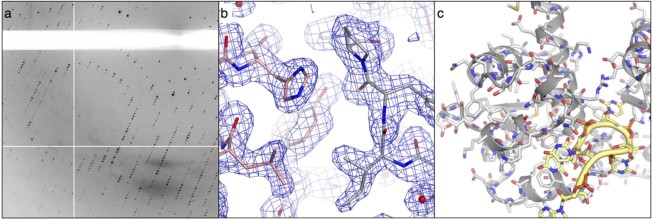
Protein crystallography: (a) detail of the X-ray diffraction pattern from a protein crystal, (b) an electron density map, with accompanying atomic model of a protein molecule. Bonds between atoms are shown as sticks, (c) the crystal structure of a protein-RNA complex. Protein and RNA chains are represented by ribbons and tubes respectively. Amino acids and nucleotides are also shown in stick representation (Colour images are available in the online version of this article).

The early ‘visualizations’ of atomic structure were limited both by the time-consuming nature of the lengthy calculations and the difficulty of representing three-dimensional information on paper (although 3D models were constructed for lecture demonstrations). With modern computers the Fourier calculations needed to produce electron density maps from diffraction data are performed in seconds and can be displayed and manipulated in 3D in real time, greatly facilitating the construction of atomic models (Figure 1).

The fact that many chemical compounds crystallize readily — often as a final step in purification — stimulated the adoption of X-ray crystallography by chemists in the 1920s. However, the development of reliable methods for growing crystals of protein molecules, which are larger, more flexible and generally depend for their structural integrity on being kept in an aqueous environment, has proved difficult. Protein crystals had been observed as early as 1840 but a key breakthrough for structural biology was made in 1934 when Bernal and Crowfoot photographed clear X-ray diffraction patterns using crystals of the digestive enzyme pepsin that, critically, had been maintained in a humid environment by sealing them in fine glass capillary tubes rather than allowing them to dry out. For the first time, protein crystals were observed to diffract X-ray beams through large angles, which meant that ultimately it should be possible to work out the protein structure at atomic resolution. This was as much a conceptual advance as a technological one. It helped to demolish colloidal theories that envisioned proteins as loose heterogeneous aggregates because the order within the diffraction pattern inferred that the pepsin molecule had a clearly defined conformation (Bernal and Crowfoot 1934). In other words, there was a structure for crystallographers to go after.

The technical difficulty of applying Fourier methods to solve the structures of complex protein molecules, which largely consisted of tackling the so-called phase problem [to work out the phase of each diffracted ray (see Brooks-Bartlett and Garman, this issue)], meant that the first crystal structures of protein molecules did not appear until the late 50s and early 60s. Watson and Crick’s 1953 structure of DNA actually beat the protein crystallographers by some years. Modelled using sparse diffraction data from the Kings’ group, their DNA model was a tour-de-force of molecular and biochemical intuition — a highly educated guess that, thanks to the symmetry and simplicity of the double-helical structure, bypassed the phase problem and turned out to be correct.

The explanatory power of the structural analysis of DNA, which immediately indicated the copying mechanism, was no less evident in the first protein structures. It was immediately apparent, for example, that the fold of the protein chain in myoglobin, an oxygen-storage molecule extracted from sperm whale muscle, was nearly identical to the α- and β-chains found in horse haemoglobin, an oxygen transporter. This revealed a striking evolutionary relationship and helped to establish evolutional biology as a molecular science. The crystal structures of the first enzymes, lysozyme and chymotrypsin (which cut up carbohydrate and protein molecules respectively), provided valuable mechanistic insights, inspiring new rounds of biochemical and structural investigation to determine how these enzymes interact with the molecules that they modify. The structure of DNA might have allowed us to decode the language of the genes but as the crystallographic analysis of proteins revealed the molecular machinery of nature in three-dimensional detail we also began to see how that language was translated into action.

To this day biological crystallography remains the pre-eminent method for determining macromolecular structures at atomic resolution and shows no real sign of slowing down (Garman 2014). The productivity of the technique has been maintained by technological developments at all stages of the process. Recombinant DNA methods now facilitate the production of protein molecules that are not naturally abundant and allow them to be engineered to optimize the prospects of crystallization. Liquid-handling robots miniaturize crystallization experiments to expand the search for solution conditions needed to grow crystals. Brighter synchrotron X-ray sources equipped with microfocus beamlines, and cryo-coolers to freeze and protect crystals from radiation damage now allow data to be gathered rapidly from crystals as small as 0.005 mm that would have been discarded as unusable ten years ago. Photographic film has been superseded by a succession of electronic detectors — the latest generation, solid-state hybrid pixel detectors, can capture more than one hundred diffraction pattern images a second (Garman 2014).

Alongside these hardware developments there have also been significant theoretical advances, including the implementation of multi-wavelength anomalous dispersion (MAD) as an effective phasing method. Once seen as technically daunting, MAD has become the experimental phasing method of choice, thanks to the worldwide growth of tunable synchrotron sources and the development of effective analytical software (Hendrickson 2014). The advent of more robust statistical procedures for X-ray data processing and refinement of atomic models has been no less important since they have helped to minimize the occurrence of errors (Kleywegt and Jones 1995) — a perennial concern for any technique that relies on indirect visualization — and even, on occasion, to root out scientific fraud (Borrell 2009). Perhaps the most remarkable computational innovation is the rise of automatic model-building procedures (Badger 2003), which have reduced the hours crystallographers spend in front of molecular graphics screens trying to wrestle bonded atoms into tubes of electron density.

The field remains highly productive: around 10,000 new crystal structures are added to the Protein Data Bank (www.rcsb.org) each year and the tally passed 100,000 in 2014. It has also greatly matured. Technical and computational automation, and the migration of crystallographic software to the web (Morin *et al.* 2013), have put crystallographic techniques almost within reach of the non-specialist, at least for straight-forward structural targets (Wlodawer *et al.* 2013). In part this movement has been propelled by the excitement that erupted around structural biology in the wake of the publication of the human genome sequence at the turn of the millennium. Once seen as a questionable and near impossible goal, the rapid development of DNA sequencing technologies surprised many and allowed the first-draft sequence to be published much earlier than predicted. With all the gene sequences in hand, some were prompted to ask whether all the structures might now be solved and structural genomics was born. It had the broad aims of characterizing all possible protein folds and generating a structural database to stimulate the search for new drugs, many of which are chemical compounds that bind to and interfere with protein function. Funding was forthcoming from public and private sources and projects sprang to life in North America, Europe and Japan in the first decade of the twenty-first century — now loosely coordinated under the aegis of the International Structure Genomics Consortium.

This quasi-industrialization of protein crystallography brought many benefits. It produced thousands of new structures (over 13,000 as of Feb 2015 — 12.4 per cent of the total) and significantly reduced the cost per structure determination to about $60,000 (Terwilliger *et al.* 2009). This international effort has also helped to validate and standardize methods at all stages of the procedure — expression, purification, crystallization, and structure determination — and stimulated the spread know-how, technology and reagents to smaller laboratories, boosting their productivity. It has clearly contributed to our understanding of biology and biochemistry at the molecular level, and stimulated the continued growth of computational structural biology, a broad discipline that aims to exploit known macromolecular structures to predict the structures and interactions of those that remain unknown (Samish *et al.* 2015). Tangible therapeutic benefits that can be attributed to structural genomics have yet to emerge but that likely reflects the fact that the determination of the structure of a potential drug target remains an early step in the very complex process of identifying, characterizing, and optimizing new drugs before they can be brought to market.

## Neutron crystallography

Biological crystallography is not confined to X-ray experiments. It can also be performed with neutrons, generated either from specially modified nuclear reactors, or spallation sources in which accelerated protons are used to knock neutrons from heavy metal targets. Though most commonly thought of as particles, wave-particle duality means that beams of neutrons will produce diffraction patterns when they pass through protein crystals.

Neutron and X-ray crystallography are complementary since neutrons interact with the nucleus at the heart of an atom while X-rays scatter primarily from the electron clouds that surround them (Lakey 2009). This complementarity is sharpened because neutrons interact most strongly with hydrogen atoms, which, with just a single electron, are difficult to detect by X-ray methods. The ability of neutrons to detect hydrogen atoms is enhanced by the very different neutron scattering properties of hydrogen and its heavier isotope, deuterium, which can be incorporated relatively easily into proteins (Blakeley *et al.* 2008). Neutron crystallography is therefore particularly useful for biochemists seeking to track the positions of hydrogen atoms as they are moved around in enzyme-catalysed metabolic reactions.

The technique does not challenge the supremacy of X-ray crystallography for protein structure determination because neutron beams are relatively weak; data collection requires very large crystals (at least 0.1 mm^3^ in volume, compared to 0.0000001 mm^3^ for X-ray experiments), days rather than minutes of beam time, and is restricted to macromolecules smaller than about 0.02 µm (Blakeley *et al.* 2008). However, it can still provide atomic-resolution information and is firmly established as a method to probe the reaction mechanisms of many different classes of enzyme for which X-ray crystal structures have already been determined, such as proteases, esterases, isomerases, and reductases, all of which catalyse the exchange of hydrogen atoms in their manipulation of the hydrogen economy of the cell. It remains an important but somewhat niche technique — most working protein crystallographers will not have strayed from X-ray sources. Nevertheless, thanks to recent increases in the intensity of neutron sources, the sensitivity of detectors, and data processing software, neutron crystallography looks set to maintain a valuable role in structural biology (Blakeley *et al.* 2008).

## Solution scattering

The application of X-ray or neutron crystallography to investigate structure demands that the biological macromolecule under investigation be crystallized, but despite the advances made over the past fifty years, this remains a hurdle at which many projects fall. However, it is possible to obtain structural information on molecules in the solution state — without crystallization — using small-angle scattering (SAS) techniques. X-ray (SAXS) and neutron (SANS) variants of SAS are both available. As the names of these methods imply, the incident beams are only deflected though small angles and the information obtained is therefore of relatively low resolution. This degradation occurs because, freed from the serried ranks imposed by crystallization, molecules tumble rapidly in solution and present themselves to the interrogating beam in all possible orientations; the resulting diffraction pattern, instead of consisting of defined arrays of spots is smeared by averaging into a fuzzy disc, bright in the middle but fading towards the outer edge. Because this diffraction disc is circularly symmetric, the recorded scattering profile varies only in one dimension — along the radius.

The information loss as a result of the averaging is certainly troubling. Nevertheless, experiments performed through the 60s, 70s and 80s — notably on the structure of the ribosome (Moore *et al.* 1986), the protein synthesis factory of the cell — showed that it was possible to retrieve three-dimensional information from one-dimensional SAXS and SANS data. The technical difficulty of these early experiments meant there were few adopters so the rate of output has lagged far behind that of crystallography. But that situation is changing.

Within the past decade or so, new computational methods for using solution scattering data to guide the construction of dummy atom models that reliably reconstruct overall molecular shapes have boosted the utility of the method (Svergun 2010, Rambo and Tainer 2013). This has re-invigorated SAS techniques and overcome some of the scepticism of more traditional structural biologists (Nagar and Kuriyan 2005). Uptake of SAS methods has been further boosted by the improvements in synchrotron and neutron sources and detector technology noted above. Liquid-handling technology now allows SAXS beamlines to run in high-throughput mode using samples of just a few microliters at concentrations as low as 1 mg/mL (Rambo and Tainer 2010).

SAXS and SANS data — and the resulting molecular models — can resolve features as fine as 10 Å, which falls well short of providing atomic detail, but increasingly these methods are being applied to discern the overall shapes of biological macromolecules (Skou *et al.* 2014). They are particularly effective for analysing the structures of complexes of two or more molecules, particularly if high-resolution structures of the components are already known. These composite structures can be assembled within the shape envelope determined by SAXS or SANS. An impressive recent example used a combination of SANS and NMR (see below) to reveal the structure of a large (390 kD) protein-RNA complex that modifies RNA to regulate ribosome assembly (Lapinaite *et al.* 2013).

Although SAXS provides more precise measurements, SANS has a particular advantage in the analysis of complexes, since selective deuteration of components within a complex and contrast matching by adjusting the H_2_O/D_2_O mix in the surrounding solvent allows their position and shape within the complex to be identified (Lakey 2009). Though less commonly applied, the contrast-matching trick can also be performed in SAXS using sucrose or heavy atoms to distinguish different macromolecular components in large complexes, such as protein, nucleic acid or lipids (Rambo and Tainer 2013, Chen *et al*. 2014).

Release from the demands of crystallization may exact a cost in resolution, but solution scattering methods benefit from being unrestricted by macromolecular size or solvent conditions. While crystallographers often require high concentrations of salts or organic compounds to grow crystals, solution scatterers are at liberty to vary the solution conditions and in particular to seek a more physiological environment for their samples.

Freedom from the constraints of the crystal lattice has further advantages since solution methods are emerging to permit the analysis of the flexibility of biological molecules. Crystallization typically ‘freezes’ a macromolecule into a single conformation. This locking of the structure is necessary for the growth of crystals that diffract to high angles and high resolution but can be problematic. In the worst cases it may give rise to misleading artefacts if molecular contacts within the crystal lattice distort the molecular structure (Krissinel 2011, Demo *et al*. 2014). Such effects are relatively rare and should not be overstated. A more common problem is the reduction or perturbation of the natural flexibility of biological molecules that is often critical for their function. Crystallography does not provide a ready remedy for the loss of dynamic information unless alternative conformations can themselves be stabilized and crystallized, for example, through interactions with binding partners, substrates or inhibitor molecules. However, in a solution that can be adjusted to reflect physiological salt concentrations, pH and temperature, biological macromolecules are likely to exhibit normal flexibility. With care solution scattering methods may be used to identify the ensemble of conformations adopted during functional operations (Rambo and Tainer 2013). As a case in point, SAXS has been used to analyse the structural changes induced by adenosine triphosphate (ATP) — the chemical fuel of the cell — to control the repair of double-strand breaks in DNA by a large ATPase-nuclease complex (Williams *et al*. 2011).

## Nuclear magnetic resonance spectroscopy (NMR)

NMR is another solution-based structure-determination method, though one that does not involve scattering of incident beams. In fact, the method relies on an entirely different physical principle: the behaviour of atomic nuclei with half-integer spin in a strong magnetic field. The spin of such nuclei, which include the common proton (^1^H) and isotopes of elements that can be incorporated in significant numbers in biological molecules (e.g.^13^C, ^15^N), makes them behave like miniature bar magnets. As such they can adopt one of two preferential orientations when an external magnetic field is applied, a low energy state aligned with the field or a slightly higher energy state aligned in the opposite direction. The energy difference between these states depends on the atomic species and on the strength of the external magnetic field: stronger fields increase the energy gap. An electromagnetic pulse of the appropriate energy — typically in the radio frequency range — can excite a nucleus to the higher state, which can then relax by the emission of a pulse of the same frequency. This nuclear resonance behaviour in a magnetic field gives the technique its name. Modern NMR spectrometers immerse biological samples in high magnetic field and are configured first to excite across a wide range of radio frequencies and then to detect the pulses emitted by resonating nuclei.

The key to determining molecular structures by NMR lies in the fact that the external field induces currents within the electron clouds that surround every atom, generating local magnetic fields that perturb the net field ‘sensed’ by each individual nucleus in a molecule. This modulates the energy gap between the high and low spin states and gives rise to small perturbations in the resonance frequency, known as chemical shifts. Chemical shift measurements provide the raw material for structure determination by NMR because the shift of each nucleus is exquisitely sensitive to particular features of its local structure, such as the proximity in space to other nuclei and the geometry and types of covalent bonds through which it is connected to its nearest neighbours in the molecule. Painstaking analysis of the structural clues within chemical shifts allows the overall structure to be determined.

Chemical shifts are extremely small — typically only 10–100 parts per million — but they are readily measured in modern instrumentation with super-conducting, high-field magnets (around 20 Telsla), which can resolve hundreds of resonances from just 100 µL of protein at 50 µM. NMR also has the advantage that spectral resolution is enhanced at higher temperatures so proteins and other macromolecules can be examined at physiological temperatures.

**Figure 2:**
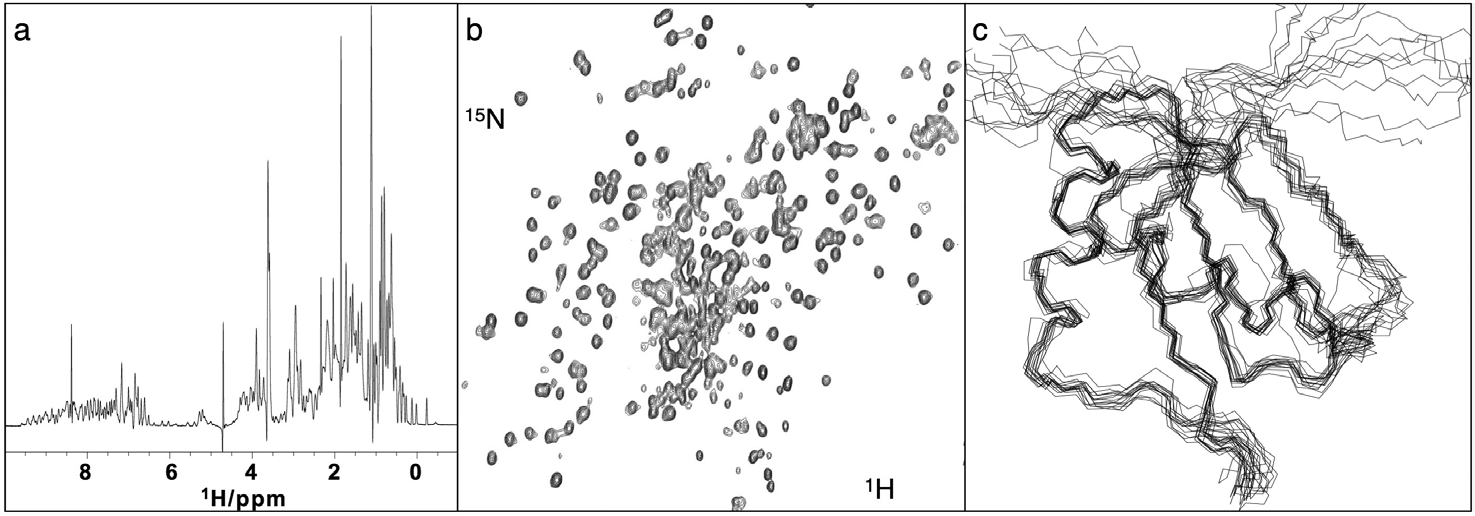
NMR: (a) a 1D spectrum showing the proton (1H) chemical shifts recorded for a protein molecule, (b) a 2D spectrum showing the correlation of NMR chemical shifts between bonded 1H and 15N nuclei in a protein structure, (c) a best-fit ensemble of protein structures calculated from NMR data; for clarity only the protein backbone is shown.

Although the physical principles underlying NMR are relatively simple, putting them into practice is a complex business and no attempt will be made here to give detailed description (Kwan *et al.* 2011, Marion 2013). What is particularly striking about the method is the indirectness of the process of information extraction. A one-dimensional (1D) NMR spectrum of a protein, which typically simply captures the frequencies at which its H atoms resonate, gives a good idea of the dispersion of chemical shifts due to the distinct stereochemical environments of each nucleus (Figure 2a). For protein structure determinations chemical shift measurements are made in 2D experiments (Figure 2b), where the magnetization excited in one nucleus can be transferred to another of a different atomic species (e.g. H-N or H-C), or 3D experiments where the transfer is between three distinct species, most commonly H, C, and N.

Acquiring and analysing NMR data for a protein molecule is an interleaved two-step process, first to assign the resonances to atoms in the amino acids known to be in the protein sequence and second to gather information on the magnitudes of the interactions between nuclei, through their spins, which can be used as structural restraints in the calculation to determine the overall structure. Assignment is typically performed in so-called triple resonance experiments in which the magnetization is transferred between H, C, and N nuclei that are closely linked by covalent bonds; analysis of the spectra derived from such transfers ultimately allows resonances to be mapped using their covalent connections all the way through the polypeptide chain. The second stage involves analysis of spin-spin interactions between pairs of nuclei, either through bonds (J-coupling), which provide information on angular relationships between sub-sets of bonded atoms, or through space [nuclear Overhauser effects], which provide estimates of the proximity of nearby atoms (up to 5 Å away). These data are combined with chemical shift information (which can signal the presence of secondary structural elements within proteins such as α-helices and β-sheets) and used to restrain molecular dynamics calculations of a 3D structure. Typically, given the uncertainties in the analysis, the results are presented as an ensemble of the ten or twenty structures that best fit the accumulated data (Figure 2c). With enough restraints the structures can be comparable in quality to an X-ray crystal structure determined at 2–3 Å resolution, which is sufficient to give atomic details (Kwan *et al.* 2011).

The complexity and indirectness of the method is such that the first protein structure solved by NMR in 1985 was greeted sceptically by crystallographers, some suspecting that information might have been cribbed from an extant crystal structure (Marion 2013). However, these doubts were soon swept away and the method is now well established. The rate of structure determination by NMR is never likely to challenge X-ray crystallography. Despite the advent of cryo-cooled radiofrequency probes and higher field magnets to boost the sensitivity and resolution of the data, signal strength drops as the molecular weight increases and routine structure determinations are still limited to proteins of no more than 25 kD.

That said, it is technically possible to determine structures up to at least 80 kD (Tugarinov *et al.* 2005) or, more commonly, to use NMR to analyse structural changes in very large complexes for which there is an existing atomic structure — such as the multi-subunit 900 kD chaperone, GroEL-GroES (Fiaux *et al.* 2002). With careful handling, the technique can be also used to solve the structures of membrane proteins, either solubilized in detergent micelles or using solid-state NMR (Bieri *et al.* 2011).

NMR therefore remains an important weapon in the structural biological armoury. Its versatility makes it a valuable complement to other techniques. For example, not only does NMR permit the determination of high-resolution structures of macromolecules that have refused to crystallize, but it can also be brought to bear on the analysis of the significant numbers of intrinsically disordered proteins that are never going to line up in a crystal lattice (Marion 2013). The locality of much of the structural information gathered in NMR experiments, caused by the fact that each nucleus only reports on its spatial relationships to nearby nuclei, can give rise to problems in structure determination: errors can be made if resonances are assigned to the wrong nuclei (Werner *et al.* 1997). However, in many applications this local reporting is a feature and not a bug because it can be used to probe protein motions that may be important for function (Bieri *et al.* 2011, Marion 2013). NMR can also readily track changes in chemical shifts that occur when a protein encounters a binding partner, such as a drug or a sequence of DNA or RNA, and so map the surface of interaction. Such chemical shift mapping may be enhanced by the judicious addition of paramagnetic functional groups to the protein or the ligand molecule. This can provide longer-range information to fix relative orientation of the binding partners and so guide the computation of docking models of complexes that have proved recalcitrant to other modes of structural investigation.

## Electron microscopy

Wave-particle duality means that, like neutrons, fast-moving electrons also behave as waves. However, in contrast to neutrons, these lighter, charged particles can be deflected in electric and magnetic fields and so it has proved possible to configure electromagnets to act as lenses that can focus electron beams. Combinations of such lenses to create microscopes raised direct imaging in biology to a new level of resolving power in the 1940s and 1950s (Masters 2009). Modern transmission electron microscopes (TEMs), the type of instrument most commonly used to examine biological samples, accelerate electrons through voltages of 200 kV or more, which gives them a wavelength of around 2 pm, or 0.02 Å, far smaller than the 1.4 Å diameter of a carbon atom. In theory, this should provide images of spectacular resolution. In reality the technique falls far short, though from the beginning it easily out-performed light microscopy by producing images of cell interiors with features as fine as 200 Å (20 nm) (Masters 2009).

**Figure 3:**
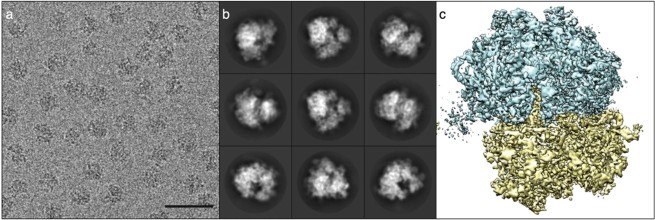
Cryo-EM: (a) a cryo-electron micrograph of bacterial ribosomes, (b) class average images derived from averaging the images of ribosome particles determined to have the same orientation, (c) a high-resolution 3D reconstruction of the ribosome. *Images are adapted from Bai et al. 2013 under the terms of a Creative Commons 3.0 Attribution Licence*

The loss of resolving power is due in part to the imperfections of electromagnetic lenses — the best ones are comparable to ‘using a the bottom of a Coca Cola bottle as a magnifying glass’ (Williams and Carter 2009). But it also arises as a result of the damage inflicted on biological samples by the beam, which means that the illuminating doses of electrons have to be kept to a minimum, a constraint that makes for noisy, low-contrast images (Masters 2009). Electron micrographs are grainy and monochrome and, to the untutored eye, somewhat reminiscent of the images beamed back to Earth from planetary probes before the advent of high-resolution digital photography (Figure 3a). There is also another price to pay for the resolution gains of EM: because of the strong interaction of electrons with matter, electron microscopes require high vacuums to prevent scattering of the beam by air molecules, an environment not well suited to the study of living systems. Only samples that are dried or preserved by cryo-cooling can be examined.

Positive and negative staining techniques were developed early on to improve the contrast of TEM images of cellular structures. Heavy metal atoms were added to cellular or molecular samples, which were then imaged after drying out as a thin layer on a transparent carbon film. Images formed in this way are a projection, a type of semi-transparent shadow, which flattens the 3D information into two dimensions.

Nevertheless, the image quality obtained with negative staining was good enough to permit the development of techniques to recover 3D information from 2D images, which was first done for highly purified preparations of virus particles in the late 1960s. Because the particles are oriented randomly — there is no crystal lattice here — each micrograph typically contains an enormous number of different views of the same 3D object. By working out the orientation of each imaged particle, the 2D information in the micrograph can be reassembled to give a three-dimensional picture. Mathematically this entails another application of Fourier methods and can be compared to doing crystallography in a computer. Crystals amplify the signal-to-noise ratio in diffraction experiments because the ordered lattice allows the scattering from each molecule to be summed coherently; with 3D reconstruction of EM images of particles, the summation is done *after* the data are collected (Figure 3b).

The first 3D image reconstruction of virus particles was a major step forward but the resolution was limited to around 70 Å because of artefacts introduced by the heavy atom staining techniques, which often damaged delicate biological samples (Crowther *et al.* 1970). These problems were circumvented by the introduction of cryo-electron microscopy (cryo-EM), which abandoned heavy metal staining in favour of flash cooling samples in liquid ethane to around -90°C. Cryo-EM preserves the samples in an extremely thin layer of glassy ice under more physiological conditions — there is no drying or exposure to heavy metals (Dubochet and Stahlberg 2001). The improved preservation comes at the cost of lower-contrast but this has largely been overcome though better control of imaging (e.g. using defocusing techniques) and by incorporating greater numbers of particles in reconstructions to improve signal-to-noise ratios.

The 3D reconstruction of the structure of Semliki forest virus by cryo-EM, reported in 1986, had a resolution of 35 Å; by 1997 the α-helices of core particles of hepatitis B virus had been resolved in a 7.4 Å reconstruction; within another decade, the resolution limit achieved with virus particles had surpassed 4 Å, allowing the polypeptide chains to be traced for the first time and putting the method within touching distance of atomic resolution (Bai *et al.* 2015).

The high symmetry of virus particles simplifies the orientation problem in cryo-EM reconstruction. The technique has also been applied to asymmetric particles such as ribosomes. With asymmetric particles, determining the orientation of individual particles in grainy images is more difficult: resolution limits have until recently been stuck at around 7 Å. On occasion the subjective bias in micrograph analysis has even resulted in conflicting structures of the same object from different laboratories — as in the case of the IP_3_R1 calcium-release channel (Murray *et al.* 2013).

However, within the past couple of years there has been a huge leap forward in the resolving power achieved in cryo-EM structure determinations (Bai *et al.* 2015). Improved statistical analysis of imaged particles now permits automatic and more objective identification of particle orientations, and filtering of conformational variants before averaging, which has significantly enhanced the quality of reconstructions. The advent of direct electron detectors has provided a further boost. They are about five times more sensitive than the CCD detectors they replaced and can record images in movie mode (at over 400 frames a second). This latter feature has solved a major problem that has only recently been identified: beam-induced sample movement (Campbell *et al*. 2012). The accumulation of charge due to bombardment with electrons causes the thin vitreous ice samples to become slightly domed while images are being recorded. However, by capturing individual frames as the sample distorts and the particles shift in position, corrections can be made to eliminate the blurring effect of this movement (Bai *et al.* 2015).

As a result of these innovations, the past two years has witnessed a torrent of new cryo-EM structures of symmetric and asymmetric particles at close to 3 Å resolution, long regarded by crystallographers as the threshold at which atomic details can be resolved and modelled reliably (Figure 3c). Indeed, during the writing of this review cryo-EM structures of bacterial ribosomes and proteasomes at better than 3 Å were reported for the first time (Campbell *et al.* 2015, Fischer *et al.* 2015). Though they have lived long in the shadow of the high-resolution capabilities of X-ray crystallography and NMR, electron microscopists are now setting out to investigate the atomic and molecular details of targets untouched by other structural biologists — complexes too fragile to be crystallized or too big to analyse by NMR. Cryo-EM may still be limited to complexes that are at least 200 kD in molecular weight, since they have to be large enough to be discerned within micrographs, but the terrain over which crystallographers and NMR spectroscopists used to range unchallenged is shrinking.

The territorial claims of cryo-EM are expanding in other directions because as well as analysing purified preparations of particles the technique can also be used to image the molecular landscape within cells. In fact, 3D reconstructions of the cellular interior can be made by combining a series of images as a sample containing frozen cells is tilted or rotated within the microscope, a technique known as cryo-electron tomography (cryo-ET) (Lučič *et al.* 2013). At present, the technique is confined to samples less than 1 µm thick, which is sufficient for imaging entire cells of some species of bacteria. Animal and plant cells are much larger and not sufficiently transparent but the development of focused ion-beam milling to isolate 1 µm slices through such cells means that tomography can peer into them as well, albeit section by section.

Contrast remains a problem, since doses have to be limited to allow all the images in a tilt series to be recorded from a single sample, but cryo-ET is providing revelatory views of biomolecules in their native environment. The information content of these images can be further enhanced by computational fitting of high-resolution 3D reconstructions (where they exist) of individual components identified within the cell, such as ribosomes and proteasomes (Brandt *et al.* 2010, Asano *et al.* 2015). Cryo-ET can also be applied to perform 3D reconstructions of complexes that are too delicate or too short-lived to be isolated for standard cryo-EM, such as the nuclear pore complex and bacterial flagellar motors (Lučič *et al.* 2013).

Direct imaging is not the only way in which cryo-EM is putting pressure on X-ray crystallographers. The electron beams within electron microscopes can also be used for diffraction experiments, as was first demonstrated thirty years ago with bacteriorhodopsin, a membrane protein that can form 2D crystals within lipid bilayers (Henderson and Unwin 1975). The technique remains somewhat niche, perhaps because of more rapid improvements in the techniques for growing 3D crystals of membrane proteins for X-ray crystallography, but has enjoyed a resurgence of late. By exploiting the stronger interactions made by electrons with biological matter, Gonen and colleagues have recently demonstrated that high-resolution protein structures can be determined by electron diffraction from crystals as small as 1 µm (Shi *et al.* 2013). It remains to be seen how widespread this new method of micro-electron diffraction will become, especially as improvements in micro-focus synchrotron beamlines and the arrival of X-ray free-electron lasers may well bring crystals of this size within the reach of more conventional crystallography (Garman 2014).

## Super-resolution microscopy

Light microscopists have returned to the structural biology fray in recent years with new ‘super-resolution’ techniques for imaging cells with visible light. These include methods such as stimulated emission depletion microscopy (STED) and photoactivated localization microscopy (PALM) (Habuchi 2014). The names hint at the sophistication of the techniques, the details of which are beyond the scope of this review. Suffice to say that these new methods rely on fluorescent tagging of molecules and sophisticated control of the patterning and timing of illumination during image capture and have achieved resolutions of around 20 nm (200 Å) in 2D and 3D. The light microscope is being rebranded as the nanoscope (Nieuwenhuizen *et al.* 2013) and the excitement in the field at these innovations has been heightened by the award of the 2014 Nobel prize in chemistry to their pioneers — Stefan Hell, William Moerner and Eric Betzig.

Super-resolution microscopy has significantly sharpened our view of cellular substructures and organelles. It is still some way shy of cryo-ET in terms of resolution but has the ability to image eukaryotic cells and, crucially, can do so while they are still alive. The dynamic processes of cellular life are now hoving into view at a resolution that would no doubt delight Abbe. Our view has yet to achieve molecular, never mind atomic resolution but that is definitely the direction of travel. A new technique known correlative cryo-electron tomography and optical microscopy (CLEM) first records images of live cells by microscopy and then flash freezes them in situ so that cryo-electron tomography can be performed. Combining the images derived from the two methods provides the best of both worlds, by delivering high-resolution snapshots of the living cell (Zhang 2013).

## Destination — reflections on our strange visions of the landscape of life

The long journey of structural biology, which started when the Braggs encouraged their co-workers to direct the penetrating power of X-rays towards biological specimens and still continues today, can rightly be described as a fantastic voyage. As I hope this review shows, the hard work to overcome the limits of human perception and turn biology into a molecular science has been full of pragmatic ingenuity.

That journey forms part of a wider transformation of perception that was a defining feature of twentieth-century science. The world may be convincingly real to our senses but the harder we have tried to reach beneath its surfaces, the more the underlying reality has receded from our grasp. Such is the hard lesson of quantum mechanics. To dig into the atomic and molecular sub-strata of life, we have had to resort to the indirect methods of crystallography, of X-ray and neutron scattering, and of NMR and electron microscopy. Each offers up 3D images of the molecules under investigation but the indirectness of the methods raises a slightly uncomfortable question: what are we actually *seeing*?

Light microscopy had its naysayers in the early days. Lady Margaret Cavendish was deeply distrustful and declared it in 1666 to be an art that ‘doth more easily alter than inform’ (Ball 2013). The same charge might easily be laid at the feet of structural biology. The images and models it produces are all, in the end, computer-generated. They are false-coloured, with atoms rendered schematically as sticks or, for more ‘realism’, as spheres. Unavoidably, these pictures are to some extent fake or, to put it more charitably, imagined. Is that a problem?

Few complaints are heard. Indeed life scientists who do not work in structural biology often express an excess of admiration for those who have grappled with its complexities and rarely question their results. Trivially this may be because none of us has ever seen a protein molecule with our own eyes so there are no reference points for what they are supposed to look like. More importantly, the images and models produced by structural biology have earned our trust because, except in rare cases of error or fraud, they are consistent with a large body of biochemical and biological data. Time and again structural biology has been recognized as possessing great explanatory power, giving us visions of the atomic details of biological macromolecules that provide deep insights into how they are synthesized and fold, how they bend and flex, and how they interact with one another in performing their cellular and extra-cellular duties. Structural biology is also at the heart of drug development and underpins the design of proteins with novel functions and even of new biomaterials.

**Figure 4:**
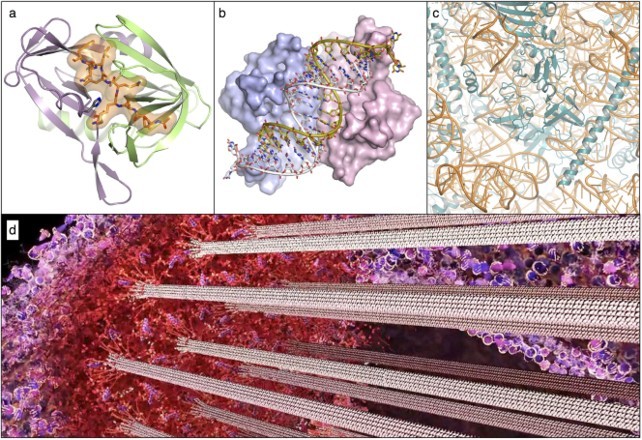
Molecular structures and the molecular sociology of the cell: (a) interaction between the foot-and-mouth disease 3C protease and a peptide it is about to cleave (PDB ID 2wv4) (Zunszain et al. 2010), (b) structure of tomato aspermy virus protein 2b bound to and neutralizing the antiviral effect of a cellular double-stranded RNA molecule (PDB ID 2zi0) (Chen et al. 2008), (c) close-up view of the ribosome, the gigantic RNA-protein machine that synthesizes proteins found in all cells (PDB ID 4v88) (Ben-Shem et al. 2011), (d) detail from a still from the animation ‘Chromosome and Kinetochore’. (https://youtu.be/0JpOJ4F4984) showing the kinetochore and associated microtubules, a complex system of molecules that separates duplicated chromosomes just prior to cell division.

The wonder and value of the endeavour is hardly in doubt but, having broken through to an invisible world beyond the barrier of the senses, the landscape revealed is also somewhat alien. It can be difficult to get your bearings. After sixty years of work that landscape is strewn with thousands of molecular structures. Most are isolated examples since the available techniques require us to use purified samples to pick off the structures one-by-one. Each is a thing of beauty, replete with significance, to the scientists who have laboured to work out the atomic details (Figures 4a–c), but the piecemeal approach makes it difficult for the uninitiated to get a sense of context and meaning. Even the initiated, absorbed in the task of determining each new structure, can sometimes forget to consider the big picture.

That picture is slowly coming into focus as the techniques of structural biology are increasingly being combined to give us a more holistic view of the molecular architecture of the cell. We are still some way short of having a complete view of what has been called the ‘molecular sociology’ of life (Robinson *et al.* 2007), but the coinage of that term is in itself a telling indicator of how far we have come. The knowledge base is now sophisticated enough to allow artistic scientists like David Goodsell to paint the crowded interiors of bacterial and eukaryotic cells (Goodsell and Johnson 2007), or Drew Berry to animate the molecules of life in cinematic narratives^1^ (Fig. 4d). These works, which draw heavily on science and keep artistic licence on a tight rein, are an exciting and impressive testament to the power of structural biology. Nevertheless they remain a little disconcerting, even to devotees like myself who are immersed in the discipline. We can marvel at the sophisticated molecular machinery within us but still feel unconnected — this is not how we usually see ourselves. Man as machine is a trope more often explored in science fiction than in science, but it is very much the story being told — and illustrated — by structural biology. We are just going to have to get used to it.

## Note

1 
http://www.ted.com/talks/drew_berry_animations_of_unseeable_biology
